# Advantages of pyruvate-based fluids in preclinical shock resuscitation-A narrative review

**DOI:** 10.3389/fphys.2022.1027440

**Published:** 2022-11-21

**Authors:** Fang-Qiang Zhou

**Affiliations:** ^1^ Independent Researcher, Las Vegas, NV, United States; ^2^ Fresenius Medical Care, Chicago, IL, United States

**Keywords:** fluid therapy, glycolysis, hypoxia, oral rehydration solution, pyruvate, peritoneal dialysis solution, shock resuscitation, pyruvate dehydrogenase

## Abstract

This review focuses on the innate beneficial effects of sodium pyruvate-based fluids, including pyruvate in intravenous solutions, oral rehydration solutions, and peritoneal dialysis solutions, on shock resuscitation with various animal models relative to current commercial fluids over the last two decades. Due to its superior pharmacological properties, pyruvate effectively sustains cytosolic glycolytic pathways and mitochondrial oxidative phosphorylation by restoration of redox potentials and reactivation of pyruvate dehydrogenase in hypoxia, even anoxia, and diabetes, reversing the Warburg effect and diabetic glucometabolic aberration. Pyruvate has been demonstrated to protect against multiorgan dysfunction and metabolic disturbance in numerous preclinical studies with various pathogenic injuries. The unique features of pyruvate potential clinical benefits encompass to efficiently correct lethal lactic acidosis *via* metabolically rapid consumption of intracellular [H^+^] and robustly protect multiorgan metabolism and function, particularly visceral organs in addition to the heart and brain, significantly prolonging survival in various animal models. Pyruvate protection of red blood cell function and preservation of the partial pressure of arterial oxygen should be highly concerned in further studies. Pyruvate is much advantageous over existing anions such as acetate, bicarbonate, chloride, and lactate in commercial fluids. Pyruvate-based fluids act as a therapeutic agent without causing iatrogenic resuscitation injury in addition to being a volume expander, indicating a potential novel generation of resuscitation fluids, including crystalloids and colloids. Pyruvate-based fluids have an enormous potential appeal for clinicians who face the ongoing fluid debate to readily select as the first resuscitation fluid. Clinical trials with pyruvate-based fluids in shock resuscitation are urgently warranted.

## Introduction

Over the past two decades, pyruvate, as an anion in aqueous solutions of sodium pyruvate, has increasingly shown its unique beneficial bioactive and pharmacological features in the protection of glucometabolic pathways and multiorgan function in various pathogenic insults, such as ischemia–reperfusion injury and/or hemorrhagic, traumatic, and septic shock. Pyruvate may be the optimal expected anion, which is superior to acetate, bicarbonate, chloride, lactate, citrate and even malate, in resuscitation fluids demonstrated in various animal studies on shock treatments, although no clinical resuscitation of shock with pyruvate-based fluids has been yet investigated to date (Zhou, 2020a; Zhou, 2022). Nowadays, the debate on fluids in shock treatments is still ongoing, and the ideal fluid is quite controversial (Zhou, 2022). This review comprehensively summarized the reports on the inherent advantages of pyruvate-based fluids in preclinical shock resuscitation in the literature. The findings strongly suggest that pyruvate-based fluids may provide advantages over their current counterparts in future clinical shock resuscitation.

## Intravenous pyruvate-based fluids in shock resuscitation

### Pyruvate-based saline

*Hypertonic pyruvate saline*: Since 1999, an experienced pyruvate research team presented a series of pioneer studies, exploring the favorable effects of intravenous (IV) hypertonic sodium pyruvate (SP, 30%) solution in swine and 22.5% pyruvate in rats, both subjected to severe hemorrhagic shock. Choosing this excess concentration of SP to reach a-5 mM of blood pyruvate level in the studies was based on previously isolated perfused hearts and neuronal cell culture experiments (Mongan et al., 1999; Mongan et al., 2003; Sharma et al., 2003; Sharma et al., 2005a). The findings first demonstrated that the pyruvate solutions were more beneficial, superior to ethyl pyruvate (EP), than normal saline (NS, 0.9% NaCl) and hypertonic 10% NaCl in resuscitation from severe hemorrhagic shock, initially discovering that pyruvate *in vivo* was an alkalizer used to correct lethal hypoxic lactic acidosis with reversing glucometabolic disturbances and a potent anti-oxidative stress/-inflammation to robustly protect multiorgan function, specifically the heart and brain, prolonging survival. The beneficial effects of pyruvate mainly came from the preservation of the nicotinamide adenine dinucleotide oxidized form/reduced form (NAD^+^/NADH) ratio, reactivation of the total/active pyruvate dehydrogenase (PDH) activities in hypoxia, and inhibition of poly-ADP ribose polymerase (PARP) activation, which catalyzes post-translational modification of proteins with NAD^+^ consumption (Mongan et al., 1999; Mongan et al., 2003; Sharma et al., 2003; Sharma et al., 2005a; Sharma and Mongan, 2010), and pyruvate acted like a PARP inhibitor and restored NAD^+^ (Mongan et al., 2003; Sharma et al., 2003; Guo et al., 2011; Yako et al., 2021). Shortly, others conducted several animal studies with hypertonic pyruvate, demonstrating the protective properties of pyruvate on the brain and heart following cardiac arrest. It was displayed that a large dose of pyruvate infusion to the steady-state arterial concentration of 3.6 mM increased energy reserves (phosphocreatine phosphorylation potential) and antioxidant defenses (glutathione reduced/oxidized form, GSH/GSSG) of the resuscitated myocardium in beagles subjected to open-chest cardiac compression after cardiac arrest in 2005 (Sharma et al., 2005b). In 2008, a large pyruvate dose (2.0 M, 0.125 mM/kg/min for 60 min) was revealed to be effective in neurological recovery from brain injury following cardiac arrest-resuscitation in dogs (Sharma et al., 2008). Recently, the hypertonic pyruvate (2.0 M) was further demonstrated to preserve vital signs, protect systemic hemodynamics, and improve metabolic and acid–base disturbances by normalizing the PDH activity, lactate/pyruvate ratio, and lactic acidosis in a blast and hemorrhagic shock model (Saha et al., 2022). Furthermore, a large dose of pyruvate (1.0 M, 0.05 mmol/kg/min for 90 min) infusion illustrated a profound attenuation of brain lesion volume by 84% vs. control and a marked decrease of DNA fragmentation by 77% vs. control in an *in vivo* stroke model with an appreciable stimulation of the hypoxia inducible factor-1α-erythropoietin (HIF-1α-EPO) signal expression in 2012 (Ryou et al., 2012), which was also observed with a regular dose of oral pyruvate rehydration (see below). Furthermore, a high concentration of IV pyruvate (0.1 mmol/kg/min for over 60 min) evidenced its preservation of antiglycation defenses in cerebral protection after cardiac arrest in pigs (Scott et al., 2017). Pyruvate action as an inhibitor of methylglyoxal-induced advanced glycation end products (AGEs), which are one of the triggers in many organ complications of critical illnesses, was subsequently shown in exogenous pyruvate protection against diabetic cataract and diabetic nephropathy (Zhou, 2022). In another study of porcine cardiac arrest with a large dose of IV pyruvate (2.0 M, 0.1 mmol/kg/min for over 60 min with a plasma pyruvate level 4–5 mM), it is worth noting that despite the occurrence of transient hypernatremia after pyruvate infusion, higher blood potassium levels were rapidly maintained close to normal ranges in contrast to NS or 10% NaCl infusion apart from systemic hemodynamics and cerebral function improvements and acidosis correction (Mongan et al., 1999; Cherry et al., 2015). This advantage might be associated with the pyruvate preservation of Na^+^-K^+^-ATPase activity that transports serum 2 [K^+^] ions into the intracellular pool, while 3 [Na^+^] ions were excluded from the cytosol in each single cycle with one ATP consumption, contributing to relieve hyperpotassemia in pyruvate therapy. Again, hyperglycemia occurred and increased after cardiac arrest, but it was quickly minimized in the pyruvate group compared to NS group (Cherry et al., 2015). This phenomenon was also shown in subsequent studies (see below). Furthermore, a continuous infusion of IV pyruvate (0.1 mmol/kg/min for 6 h) during the extracorporeal membrane oxygenation (ECMO) protocol in an immature swine model confirmed that it enhanced anaplerotic flux (replenishment of tricarboxylic acid (TCA)-cycle substrates) through pyruvate carboxylation and activated fatty acid oxidation, improving mitochondrial nutrients and energy metabolisms in the immature myocardium under conditions emulating ventricular unloading during ECMO (Ledee et al., 2015). Therefore, these extremely high concentrations (2.0 M) and large doses of pyruvate infusion had been known as optimal in preclinical studies and clinical trials (Sharma et al., 2015; Zhou, 2022). Moreover, as an EP alleviation of acute pancreatitis in many studies, a high dose of IV SP (0.25 mg/kg/h for 4 h) infusion vs. NS verified its protection against cerulein-induced acute pancreatitis with a decline of hyperamylasemia and alleviation of histopathological changes in rats (Ziolkowski et al., 2008).

*Isotonic pyruvate saline*: On the other hand, a preliminary study first illustrated in 2001 that resuscitation with pyruvate saline (1.7% NaPyr, 154 mM) was superior to NS (0.9% NaCl, 154 mM) infusion (80 ml/kg) in profound hemorrhagic shock for a 60-min period, greatly enhancing the survival (90% vs. 30%) at 90 min after fluid infusion in rats (Slovin et al., 2001). Consistent with the previous findings regarding pyruvate renoprotection against oxidative stress (Salahudeen et al., 1991), it was first shown in 2001 that the IV infusion of equimolar pyruvate/chloride saline (NaCl 104 mM, NaPyr 50 mM) was discovered with many advantages over NS in renoprotection in a III-degree burn shock model of rats subjected to 50% total body surface area (TBSA) burns, according to the Parkland formula: 4.0 ml/kg/1%TBSA for the fluid volume. The results showed not only prevention from hyperchloremia but also protection of kidney function: blood hematocrit, serum creatinine level, renal tissue water content, and vascular permeability 4 h after burns were significantly increased in the NS group in comparison with the pyruvate group, whereas no significant differences of these parameters existed between the pyruvate and control groups, although kidney histopathological changes were not detected (Han et al., 2011). All these data strongly indicate that pyruvate in saline will be more beneficial than traditional NS in clinical shock resuscitation as SP avoiding iatrogenic resuscitation injury resulted from NS-induced hyperchloremia (renal vascular contraction and glomerular filtration rate decline) and providing multiorgan protection beyond both being a volume expander.

It should be noted that in an *in vitro* investigation of red blood cells (RBCs) in dogs in simulated bypass surgical procedures (Gou et al., 2012), the pyruvate/chloride saline was profoundly protective of the glycolytic ATP generation and the ATPase activity, but inhibitive of endothelial nitric oxide synthase (eNOS) in RBCs and nitric oxide (NO) levels in plasma compared to NS, apparently suggesting that the pyruvate solution protects multiple cells/tissue metabolism and inhibits inflammation throughout the whole body in both hypoxia and anoxia because of its beneficial impacts on the anaerobic glycolytic metabolism of RBCs (Gou et al., 2012). Also, RBCs are oxygen sensors that can independently to dilate the microvascular system in hypoxia *via* glycolytic ATP release to improve tissue ischemia in addition to exerting oxygen delivery. The glycolytic ATP released from RBCs then diffuses to the vascular endothelium, resulting in activation of a vasodilator mechanism (Dietrich et al., 2000). Intriguingly, pyruvate, other than lactate, is used to facilitate the favorable effects, and the latter even interferes in ATP release from RBCs in addition to the inhibition of its generation (Rozier et al., 2007). Because the Na^+^-K^+^-ATPase activity is glycolytic ATP-dependent, the glycolytic ATP plays a key role in maintaining the basic cellular structure and function, such as membrane depolarization and homeostasis of intracellular pH, throughout the cells of the whole body (Sugiyama et al., 2001; Schousboe et al., 2011). The pyruvate protection of RBCs must be of a significant clinical importance in shock resuscitation (see below).

*Pyruvate Ringer’s solution*: Simultaneously, it was first released in 2001 (the abstract form reported in Surg Forum, 1999) that a regular pyruvate dose with pyruvate Ringer’s solution (28 mM) was similarly efficient as Ringer’s ethyl pyruvate solution (REPS) in protecting from intestinal mucosal damage induced by the mesenteric ischemia–reperfusion injury in rats (Sims et al., 2001). Subsequently, pyruvate Ringer’s solution was reproducibly demonstrated of its efficacy in shock resuscitation from hemorrhagic and traumatic/burn shock in various animal models (Koustova et al., 2003; Lin et al., 2005; Flaherty et al., 2010; Gurji et al., 2013; Hu et al., 2013; Liu et al., 2016a; Hu et al., 2018). The results from these studies still further confirmed that the exogenous pyruvate in Ringer’s solution benefits in increasing arterial blood pH values and pyruvate levels and decreasing lactate/pyruvate ratios in shock resuscitation compared with the lactate Ringer’s counterpart, leading to the full correction of fatal hypoxic lactic acidosis (Flaherty et al., 2010; Hu et al., 2013). The favorable effects also manifested the improvement of redox potentials (NAD^+^/NADH and GSH/GSSG), inhibition of inflammation, and preservation of multiorgan function and even the partial pressure of arterial oxygen (*P*aO_2_), remarkably prolonging survival (Hu et al., 2013; Liu et al., 2016a). The aforeementioned results substantiated that pyruvate uniquely preserved canonical anaerobic glycolytic pathways and oxidative phosphorylation in hypoxic conditions induced by pathogenic attacks, protecting against multiorgan dysfunction and cellular apoptosis (Koustova et al., 2003; Hu et al., 2013). Moreover, pyruvate Ringer’s solution increased plasma membrane-located monocarboxylate transporter (MCT 1) levels, through which pyruvate, lactate, or other monocarboxylate imports from the extracellular to intracellular compartments with hydrogen (proton, [H^+^]) flux in symport, in endothelial cells and neutrophil and activated the astrocytes of brain in resuscitation from hemorrhagic shock in rats (Lin et al., 2005). Another important discovery was that pyruvate Ringer’s solution as a novel carrier in hydroxyethyl starch (HES) 130/0.4 was first shown with significant renoprotective properties including kidney function and histopathological aberration, as well as the preservation of intestinal microvascular permeability and barrier function [Wang XN. Thesis. 2015. doi: 10.7666/d.Y2785289.], in resuscitation of lethal burn shock in rats, compared to acetate, chloride, and lactate in counterparts of commercial HES 130/0.4 products (Hu et al., 2018). These results strongly indicate that pyruvate saline, pyruvate/chloride saline, or pyruvate Ringer’s formulation as a new carrier solution of colloids in natural plasma, albumin, or artificial plasma products and even hemoglobin-based oxygen carriers would be more protective of organ function relative to current carriers in commercial colloids in the clinical fluid therapy in the future.

High doses of IV pyruvate hold a systemic protection property, including hemodynamics, energy metabolism, multiorgan function, and acid–base balance, in critical care (Kristo et al., 2004; Kumar et al., 2020), as shown with peritoneal pyruvate in acute brain injury (Lee et al., 2001; Moro et al., 2016). However, it is worth emphasizing now that regular doses of pyruvate in saline, Ringer’s solution, or even oral fluid (see below) would be efficient as a large dose of pyruvate in clinical shock resuscitation. The maintenance of high blood concentrations to 4–6 mM with 2.0 M pyruvate infusion is not clinically required unless 11.0% (1.0 M) pyruvate infusion *via* the central veinous catheter is preferable in specific clinical conditions (Sharma et al., 2015). Additionally, a very small dose of pyruvate in the cardioplegic solution (10 mM) showed enhancement of cardiac function in 15 patients relative to the lactate counterpart (23.8 mM) during coronary artery bypass graft surgery (Olivencia-Yurvati et al., 2003). Pyruvate alleviation of myocardial injury and improved postsurgical recovery of cardiac performance were affirmed in pigs by using pyruvate-enriched cardioplegic solution (23.8 mM) (Olivencia-Yurvati et al., 2003; Ryou et al., 2010).

### Oral pyruvate rehydration solution/salt

Since the 1970s, the World Health Organization-guided oral rehydration solution/salt (WHO-ORS) has played a crucial role in the prevention and treatment of children’s diarrhea and cholera worldwide, saving millions of lives per year. Therefore, WHO-ORS was hailed as the most important medical advance in the last century by the Lancet (Editorial, 1978; Zhou, 2020a). ORS and oral rehydration therapy (ORT) with a history of over half a century has been now prevailing in fluid interventions of burns in children and even adults (Pham et al., 2008; Milner et al., 2011) because of the prompt intestinal absorption of water and salts based on physiological findings of Na^+^-Glucose cotransporter in the intestinal epithelium in the 1950s. Recently, the ORT was even successfully used in all patients of a renowned hospital in addition to patients at intensive care units (ICUs) when NS was in short supply (Patiño et al., 2018).

*Standard and low osmolar WHO-ORS*: WHO-ORS consists of four chemicals: sodium bicarbonate, sodium chloride, potassium chloride, and anhydrous glucose with the osmolarity of 331 mOsm/L (WHO-ORS I), which can be easily prepared at home; with the osmolarity of 311 mOsm/L (WHO-ORS II) by equimolar sodium citrate replacement of sodium bicarbonate; and with osmolarity of 245 mOsm/L (WHO-ORS III) in reduced-osmolarity citrate-contained ORS (Pulungsih et al., 2006; Zhou, 2020a; Zhou, 2022). Now, the last one is more popular in clinical use due to its easy absorption from the intestine and a long shelf-life.

*Pyruvate-ORS*: In 2012, a pyruvate-based ORS (Pyr-ORS) was first innovated by an equimolar pyruvate (3.5 g/L) substitute of equivalent bicarbonate (2.5 g/L) in WHO-ORS I (Chinese patent, 2012). Pyruvate in Pyr-ORS was first demonstrated with advantageous effects over bicarbonate in WHO-ORS I in enteral rehydration of severe injuries in rats following a 35% TBSA full-thickness scald in 2013 (Hu et al., 2014a). The results showed that the intestinal epithelial Na^+^-K^+^-ATPase activity, aquaporin-1 (AQP-1) expression, and intestinal mucosal blood flow (IMBF) were significantly decreased in all scald groups but significantly preserved in group Pyr-ORS relative to group WHO-ORS I. The intestinal absorption of water and sodium with pyruvate in ORS was increased by around 20% relative to the counterparts. The intestinal histopathological alterations were also more improved in the Pyr-ORS group than in the WHO-ORS I group (Hu et al., 2014a). Further studies with a rat hemorrhagic shock model uncovered that enteral rehydration with pyruvate in Pyr-ORS fully corrected fatal lactic acidosis, robustly inhibited systemic oxidative stress/inflammation, greatly increased visceral blood flow, and markedly prolonged survival, compared to bicarbonate in WHO-ORS I. The amount of administered enteral Pyr-ORS equals around 15 g/70 kg if given to a man, according to the calculation in the experiment (Yu et al., 2015). Thereafter, Pyr-ORS was compared to WHO-ORS II-contained equimolar citrate (2.9 g/L, ORS-Cit) in enteral rehydration of rats subjected with 35% TBSA burns (Hu et al., 2016). The data evidenced that pyruvate was superior to citrate in ORS in the stimulation of the HIF-1α-EPO signal pathway, preservation of IMBF and zonula occludens-1 (ZO-1, one of major intestinal barrier tight junction proteins) expression, and improvement of intestinal mucosal histological injury score at two time points during 4.5 h after scalding. The activation of HIF-1α-EPO signaling cascades may be closely associated with pyruvate efficiency and long-term pharmacological beneficial effects for at least several hours following the termination of pyruvate infusion or ingestion (Hu et al., 2016; Zhou, 2022). Further comparison was conducted in dogs that underwent 50% TBSA burn shock (Liu et al., 2016b). The results displayed that hemodynamic parameters were significantly improved in the Pry-ORS group with remarkedly lower plasma levels of vascular endothelial growth factor (VEGF) and platelet activating factor (PAF), relative to the ORS-Cit group (WHO-ORS II). Notably, severe lactic acidosis was also fully reversed in the Pyr-ORS group, instead of the ORS-Cit group (Liu et al., 2016b). Clearly, pyruvate would be superior to citrate in the standard WHO-ORS for clinical shock rehydration.

Recently, the novel reduced-osmolarity Pyr-ORS contained NaPyr 3.5 g/L, NaCl 2.0 g/L, KCl 1.5 g/L, and anhydrous glucose 13.5 g/L with the osmolarity 247 mOsm/L was further compared to low osmolar WHO-ORS III (245 mOsm/L) in enteral rehydration of severely scalded rats (Liu et al., 2018). Interestingly, the results illustrate that systemic hemodynamic indexes are considerably preserved with a higher *P*aO_2_ level in addition to improving visceral organ surface blood flow and organ (heart, liver, and kidney) function in the Pyr-ORS group. Only pyruvate-based ORS, other than citrate- (or bicarbonate-) based WHO-ORS, can correct severe lactic acidosis, although both ORSs have an equal buffer capacity: equivalent to 30 mmol/L bicarbonate, significantly enhancing survival rates (Yu et al., 2015; Liu et al., 2016b; Liu et al., 2018). It is significant in clinical settings that pyruvate, instead of current anions, such as acetate, chloride, citrate, and lactate, in resuscitation fluids specifically and efficiently corrects lethal hypoxic lactic acidosis in shock resuscitation because of its distinctive metabolic features: rapidly consuming intracellular [H^+^] by the LDH reductive reaction and gluconeogenesis in the cytosol and the oxidative phosphorylation in mitochondria on an equimolar basis in addition to its low buffering capacity with the dissociation coefficient p*K* 2.49 (Liu et al., 2018; Wang et al., 2018; Zhou, 2020a). It is worth noting that, as mentioned previously, *P*aO_2_ is sustained at a higher level in IV or enteral rehydration with pyruvate-based fluids in shock resuscitation, probably due to systemic hemodynamics and RBCs improvement (Mongan et al., 1999; Hu et al., 2013; Liu et al., 2018). In this respect, a few studies have demonstrated that pyruvate-restored RBCs can improve liver function in blood infusion and renal oxygenation in animal models and early clinical bypass surgery studies (Dennis et al., 1978; Krausz et al., 1981; Raat et al., 2009; Xia et al., 2016). Moreover, pyruvate can inhibit VEGF and PAF and protect vessel endothelium as well as RBCs dilation of microvessels (Lee et al., 2004; Gou et al., 2012; Liu et al., 2016b), facilitating improving microcirculation in hypoxia, which has a significant clinical value in shock resuscitation and needs further intensive investigations.

Alternatively, in contrast to the large dose of IV pyruvate (100 mg/kg plus 10 mg/kg/min for 90 min) protecting brain function following asphyxial cardiac arrest in lambs (Kumar et al., 2020), a small dose of oral pyruvate in Pyr-ORS also improved the brain function after asphyxial cardiac arrest during the first 24 h of experiments in rats (15 g SP if translated to a 70 kg man) (Bai et al., 2017). The pyruvate level in brain tissues and survival neurons in the hippocampal CA1 region was significantly increased in the pyruvate group vs. the control group. The results of the water maze tests of brain function were greatly ameliorated in the pyruvate group (Bai et al., 2017). In addition, oral pyruvate saline (1.7% SP) as drinking water for 2 weeks, compared to NS, significantly protected from alterations of pathological structure and function of L5 dorsal root ganglia (DRG) tissues in rats subjected to simulated weightlessness (Li et al., 2022). These data support oral pyruvate benefits in injured nervous tissues, though the dose is relatively higher in the latter investigation.

Accordingly, in the oral rehydration of clinical burn shock, Pyr-ORS should be particularly favorable with more prospective clinical benefits and tolerance (Milner et al., 2011; Gómez et al., 2018; Baird et al., 2021). Importantly, to make patients survive, unsanitized Pyr-ORS can be readily applied in prehospital rescue in ambulances and acute injuries specifically in poor resource environments with a large scale, such as earthquakes. Also, pyruvate in modified Pyr-ORS formulas can work as functional drinks but essentially acts as a first-aid medicine in specific conditions. Pyr-ORS-based functional beverages may be helpful in the prevention and treatment of diabetes in a population (see below).

### Pyruvate-peritoneal dialysis solution in adjunct peritoneal resuscitation

A new advance in experimental shock resuscitation is that adjunct/direct peritoneal resuscitation (DPR) with clinical commercial 2.5% (glucose) lactate-based peritoneal dialysis solution (L-PDS, Dianeal PD 2, 396 mOsm/L) in resuscitation from hemorrhagic shock in 2003 (Zakaria et al., 2003a; Zakaria et al., 2003b). The peritoneal resuscitation was intensively investigated by adjuvant to regular IV resuscitation or alone in animal models, and its clinical case reports also received attention recently (Smith et al., 2014; McKenzie et al., 2017; Ribeiro-Junior et al., 2022). The favorable features of DPR are mainly due to prompt and persistent visceral vasodilation *via* the improvement of endothelial function and NO generation to dilate visceral microcirculation induced by the hyperosmolarity of PDS in the abdominal cavity, appreciably reversing visceral hypoperfusion. The visceral hypoperfusion still exists even if systemic hemodynamic indexes are normalized after clinical conventional IV resuscitation. Thus, the DPR can enhance splanchnic and distant organ perfusion and promote systemic oxidative metabolism (Zakaria et al., 2003a; Zakaria et al., 2003b). Recent findings indicated that DRP reduced lung injury *via* the downregulation of multiple mRNA expressions of inflammatory mediators in rats with acute brain death (Weaver et al., 2020).

On the other hand, it was first found in 1995 that acidic (pH 5.2) pyruvate-based peritoneal dialysis solution [P-PDS, equimolar pyruvate (40 mM) replacement of lactate in commercial lactate-based PDS, L-PDS] efficiently sustained neutrophilic intracellular pH (pHi) in a near-normal range, whereas equal acidic L-PDS induced a rapid decrease to pHi 6.1 in a few minutes in pH normal human neutrophils mixtures *in vitro* (Ing et al., 1997). The result strongly evidences that pyruvate, in contrast to lactate, preserves the intracellular homeostasis of glucose metabolism and acid–base balance in human neutrophils in addition to its weak buffering capacity (Ing et al., 1997; Zhou, 2001). Consequently, advantages of pyruvate over lactate in experimental PDS were investigated, and the beneficial effects of pyruvate were further analyzed (Zhou, 2001; Wu et al., 2005; van Westrhenen et al., 2008).

Given the aforementioned findings, the first adjunct peritoneal resuscitation with 2.5% (glucose) P-PDS study was carried out in comparison with L-PDS in rats subjected to hemorrhagic shock in 2013 (Hu et al., 2014b). As expected, the DPR with pyruvate was superior to lactate in PDS in efficient preservation of blood perfusion of visceral organs: liver, kidney, and intestine of severe hemorrhagic rats. Additionally, the expression of intestinal barrier protein ZO-1 density was more accumulated in the P-PDS group than in the L-PDS group. Intriguingly, 2.2% (400 mOsm/L, equal to Dianeal PD 2) simple pyruvate solution without glucose and chemicals but with an equal osmolarity to P-PDS showed the most beneficial effects among the three test solutions (Hu et al., 2014b). Further studies verified that P-PDS also efficiently reversed hypoxic lactic acidosis and protected from intestinal histological injury in DPR, not only preserving visceral organs but also distant organs, such as the spinal cord, in rats from hemorrhagic shock, and high pyruvate concentrations in P-PDS exhibited more promising effects (Zhang et al., 2014; Lu et al., 2015; Zhang et al., 2020a; Xiong et al., 2020). Therefore, the DPR conducted with P-PDS would be a perspective in clinical settings.

Because of the prognostic severity of hypoxic lactic acidosis and a lack of clinical effective intervention in ICU patients to date (unless hemofiltration with complicated procedures and expenditure) (Broder and Weil, 1964; Gunnerson et al., 2006; García-Camacho et al., 2020), all pyruvate-based fluids in IV infusion, ORS, or DPR in regular doses that can reverse hypoxic lactic acidosis in shock resuscitation are quite appealing to clinicians who are facing the ongoing debate on fluids and may be initially selected in a variety of clinical scenarios (Zhou, 2022). The exogenous pyruvate metabolic profile in intracellular circumstances was explicitly provided previously (Hu et al., 2013; Wang et al., 2018; Zhou, 2022).

Finally, a recent preliminary finding should be concerned. With the severe scald model of rats subjected to multiple organ dysfunction syndrome, the DPR of P-PDS with 2.5% glucose still effectively preserved the islet β-cell function, as illustrated by a higher level of homeostasis model assessment of β-cell (HOMA-β), avoiding hyperglycemia (Ning et al., 2020). This result is consistent with a new conception that pyruvate which is involved in glucose-stimulation insulin secretion (GSIS) is an insulin stimulator, as demonstrated in Type 1 diabetes patients by reducing the total daily dose (TDD) of insulin injection with oral pyruvate and diabetic *db/db* mice with increasing blood insulin levels by oral Pyr-ORS treatments (Petkova et al., 2007; Inoue et al., 2016; Zhang et al., 2020b). Significantly, oral pyruvate in both 1.0% pyruvate and 0.35% pyruvate of Pyr-ORS, which concomitantly contains 1.35% glucose, as drinking water comparably rejuvenated the renal pyruvate kinase (PK) and PDH activities with a decrease in blood glucose in diabetic mice, demonstrating that a small dose of oral pyruvate can effectively reverse the Warburg effect in diabetes (Zhang et al., 2020b). In this regard, exogenous pyruvate, instead of contemporary anions, reveals another specific pharmacological property to reactivate PK and PDH activities and sustain islet β-cell function, preventing hyperglycemia in critical care patients. It is well-known that stress hyperglycemia is common in non-diabetic patients in the ICU specifically with traumatic brain injury, severe sepsis, and systemic inflammatory response syndrome. Hyperlactatemia and/or accompanying hyperglycemia is a critical causation of poor prognosis in ICU patients (Broder and Weil, 1964; Gunnerson et al., 2006; Bar-Or et al., 2019). Several prior studies supported that pyruvate protected against islet β-cell damage both *in vitro* and *in vivo* (Yates et al., 1990; Rastellini et al., 1995; Brown et al., 2005). Thus, pyruvate is ideally a stimulant of insulin secretion. Accordingly, pyruvate-based fluids have an innate unique metabolic capacity to prevent and correct hyperlactatemia, hyperglycemia, and hyperpotassemia in clinical settings, which all commercial fluids lack, in shock resuscitation.

Nevertheless, despite that varying forms of DPR have been demonstrated to attenuate visceral hypoperfusion, the DPR volume, rate, and dwell time and pyruvate concentration in administration are still a complex question in clinical settings (Hu et al., 2014b; Zhang et al., 2020a; Schucht et al., 2021).

## Pyruvate organ protection and clinical application

Mounting *in vivo* experimental and clinical findings have documented beneficial effects of pyruvate on the protection of multiorgan metabolism and function against various pathogenic attacks (Ariyannur et al., 2021; De Moraes et al., 2022; Yang et al., 2022; Zhou, 2022). Increasing evidence grounded on cytotoxic tests in animal studies and preliminary clinical trials with several hundreds of healthy volunteers and patients subjected to heart failure, cardiomyopathy, chronic liver disease, diabetes, and mitochondropathy, as well as pyruvate loading tests over the past 50 years all endorse that SP products in commercial markets are basically safe, effective, and practical in human illnesses (Mateva et al., 1996; Petkova et al., 2007; Inoue et al., 2016; De Moraes et al., 2022; Yang et al., 2022; Zhou, 2022).

The core concern that pyruvate aqueous solutions are unstable at room temperature and impurities of pyruvate products (pare-pyruvate) are cytotoxic, which were plausibly referred in EP studies (Koprivica et al., 2022), has been essentially solved (Zhou, 2022). Preliminary pharmaceutical tests including accelerated (40°C, relative humidity 75% for 6 months) and long-term stability tests of the new drug of sodium pyruvate saline produced in a GMP workshop (according to the US patent, 2014) were qualified (unpublished data). There has been no evidence that pyruvate has *in vivo* cytotoxicity in humans up to now (Zhou, 2022). Therefore, there are few obstacles to pharmaceutically produce pyruvate aqueous solutions, at least as an injection with pyruvate powder dissolved just prior to use, for clinical use. In all, as indicated previously, pyruvate holds several unique pharmacological properties, such as improvement of glucometabolic disorders with PK and PDH reactivation, enhancement of hypoxia/anoxia tolerance and redox potential, reversal of hypoxic lactic acidosis, stimulation of HIF-1α-EPO signals and insulin secretion, and inhibition of AGEs (Zhou, 2022). These favorable features, which meet the desirable properties of an ideal resuscitation fluid (Edwards and Hoareau, 2021), will contribute to develop a novel generation of clinical resuscitation fluids including crystalloids and colloids following the use of NS and lactated Ringer’s solution for a couple of centuries. It is self-evident that if pyruvate saline replaces NS and pyruvate Ringer’s solution substitutes the lactate counterpart in ICU patients, including patients with severe virus infection, such as COVID-19 and Ebola, particularly in diabetes and elderly with or without organ comorbidities (Zhou, 2020b; Zhou, 2022), the clinical courses and survival rates would be greatly improved. Conceivably, novel pyruvate-based fluids would promote the therapeutics advances of critical care medicine and enhance the social healthcare level. Nevertheless, SP has not been approved by the U.S. FDA. Intensive studies and randomized clinical trials are urgently needed with pyruvate-based fluids in shock resuscitation.

Finally, there have also been numerous studies regarding EP (lipophilic ester derivative of pyruvate sodium salt) beneficial effects on organ protection in preclinical shock resuscitation since 1999 (Sims et al., 2001; Koprivica et al., 2022). These data additionally and strongly support the advantages of a pyruvate moiety of SP in clinical fluid therapy. However, EP does not correct lactic acidosis despite that hyperlactatemia is ameliorated in a rodent model, probably because of [H^+^] generation when it is hydrolyzed to the pyruvate ion on an equimolar basis in plasma (Zhou, 2022). Furthermore, EP does not work in humans and a phase II clinical trial in cardiac surgery has failed (Bennett-Guerrero et al., 2009; Koprivica et al., 2022), further implying that sodium salt of pyruvate-based fluids in various formulas will be superior and attractive in clinical fluid therapy in the future.

## Conclusion

The novel pyruvate-based fluids including IV solution, ORS, and PDS exhibit beneficial impacts compared to current counterparts in preclinical studies on shock resuscitation. Specifically, pyruvate saline is advantageous over NS and pyruvate Ringer’s solution is superior to lactated Ringer’s solution in the protection of cell/organ metabolism and function. The pyruvate-based fluids will act not only as a volume expander but also as an ideal therapeutic agent to various pathogenic injuries in future fluid therapy. Intensive investigations and clinical trials with pyruvate-based fluids are urgently warranted in shock resuscitation. The clinical uses of pyruvate-based fluids will be of a great significance in promoting basic medical therapy in ICU patients and elevating the level of social medical care.

## Author’s note

The author is a retired independent medical researcher. The opinions or assertions contained herein are not a reflection of the view of Fresenius Medical Care, Dialysis Centers in Chicago, IL, United States.

## Abbreviations

AGEs, advanced glycation end products; DPR, direct peritoneal resuscitation; eNOS, endothelial nitric oxide synthase; EP, ethyl pyruvate; GSH/GSSG, glutathione reduced form/oxidative form; HES, hydroxyethyl starch; HIF-1α-EPO, hypoxia-inducible factor-1α-erythropoietin; [H^+^], hydrogen, proton; IMBF, intestinal mucosal blood flow; IV, intravenous; LDH, lactate dehydrogenase; L-PDS, lactate-based peritoneal dialysis solution; NAD^+^/NADH, nicotinamide adenine dinucleotide oxidative form/reduced form; NO, nitric oxide; NS, normal saline; ORS, oral rehydration solution/salt; PAF, platelet activating factor; *P*aO_2_, partial pressure of arterial oxygen; PDH, pyruvate dehydrogenase; Pyr-ORS, pyruvate-based ORS; P-PDS, pyruvate-based PDS; RBCs, red blood cells; SP, sodium pyruvate; TBSA, total body surface aera; TCA, tricarboxylic acid cycle; VEGF, vascular endothelial growth factor; and ZO-1, zonula occludens-1.
